# A different view on the Necker cube—Differences in multistable perception dynamics between Asperger and non-Asperger observers

**DOI:** 10.1371/journal.pone.0189197

**Published:** 2017-12-15

**Authors:** Jürgen Kornmeier, Rike Wörner, Andreas Riedel, Ludger Tebartz van Elst

**Affiliations:** 1 Institute for Frontier Areas of Psychology and Mental Health, Freiburg, Germany; 2 Eye Center, University of Freiburg, Freiburg, Germany; 3 Department of Psychiatry and Psychotherapy, Medical Center, University of Freiburg, Freiburg, Germany; 4 Faculty of Medicine, University of Freiburg, Freiburg, Germany; Technical University of Madrid, SPAIN

## Abstract

**Background:**

During observation of the Necker cube perception becomes unstable and alternates repeatedly between a from-above-perspective (“fap”) and a from-below-perspective (“fbp”) interpretation. Both interpretations are physically equally plausible, however, observers usually show an a priori top-down bias in favor of the fap interpretation. Patients with Autism spectrum disorder are known to show an altered pattern of perception with a focus on sensory details. In the present study we tested whether this altered perceptual processing affects their reversal dynamics and reduces the perceptual bias during Necker cube observation.

**Methods:**

19 participants with Asperger syndrome and 16 healthy controls observed a Necker cube stimulus continuously for 5 minutes and indicated perceptual reversals by key press. We compared reversal rates (number of reversals per minute) and the distributions of dwell times for the two interpretations between observer groups.

**Results:**

Asperger participants showed less perceptual reversal than controls. Six Asperger participants did not perceive any reversal at all, whereas all observers from the control group perceived at least five reversals within the five minutes observation time. Further, control participants showed the typical perceptual bias with significant longer median dwell times for the fap compared to the fbp interpretation. No such perceptual bias was found in the Asperger group.

**Discussion:**

The perceptual system weights the incomplete and ambiguous sensory input with memorized concepts in order to construct stable and reliable percepts. In the case of the Necker cube stimulus, two perceptual interpretations are equally compatible with the sensory information and internal fluctuations may cause perceptual alternations between them—with a slightly larger probability value for the fap interpretation (perceptual bias). Smaller reversal rates in Asperger observers may result from the dominance of bottom-up sensory input over endogenous top-down factors. The latter may also explain the absence of a fap bias.

## Introduction

The environmental information available to our senses is incomplete and to varying degrees ambiguous. Our perceptual system needs to disambiguate and interpret this information in order to construct fast solutions to all types of sensory input of whatever quality (the “visual inference problem”, [1]). Perception has thus been widely discussed as a weighting of sensory (bottom-up) information with memorized (top-down) concepts in terms of Bayesian probability estimation (e.g., [2]). The relative contributions of bottom-up and top-down factors during the perceptual process strongly depend on the quality of the sensory information, the relation between observed objects and their context, and the availability of memorized concepts that fit to the sensory input.

Patients with autism spectrum disorder (ASD) show patterns of altered visual processing (e.g., [3]). Their perception is described as being generally dominated by bottom-up sensory information, whereas top-down contribution seems to be underweighted. In particular, ASD patients are often oversensitive to loud noises or bright colours. Their perceptual interpretations are dominated by small sensory details, whereas they have difficulties to integrate spatial context [4] and prior perceptual experiences (e.g. [5,6]). Further, ASD observers are less susceptible to optical illusions (e.g., [7,8]). Perceptual abnormalities also belong to the core features of ASD and have been included into the diagnostic criteria of DSM-5 (www.dsm5.org*)*.

**Fig 1:**
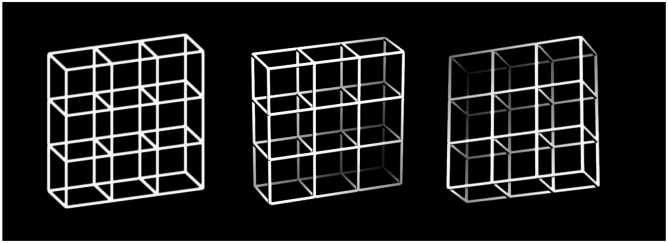
Lattice stimuli. The ambiguous Necker lattice (left), a combination of nine Necker cubes, together with disambiguated lattice variants with depth cues (middle lattice = from-above-perspective “fap”; right lattice = from-below-perspective “fbp”), representing the two most probable 3D lattice interpretations [9,10].

Ambiguous figures like the famous Necker cube (Fig 1, left, depicts the “Necker lattice”, a combination of 9 Necker cubes, [9,10]) are paradigmatic in this context. Their sensory information allows for two or more equally probable but mutually exclusive interpretations and our perception reverses spontaneously between them. Psychophysical studies on ambiguous figures typically analyse reversal rates, dwell times and dwell time distributions (e.g., [11–14]). Reversal rates are the number of perceptual reversals per time unit. Dwell times are the transient periods of stable percepts between two reversals and mean dwell times are inversely related to the reversal rates.

In the present study we used the ambiguous Necker lattice as stimulus (Fig 1, left). The Necker lattice is a combination of 9 Necker cubes and has been extensively used in previous experiments from our group (e.g., [15]). The physical information of the lattice provides equal evidence for two interpretations, the from-above-perspective (fap, Fig 1, middle) and the from-below-perspective (fbp, Fig 1, right), however, observers’ perception seems to be a priori biased in favour of a from-above-perspective (e.g., [16–21]). This finding may reflect a kind of everyday-statistics: We typically look much more often down than up on objects. The perceptual bias is reflected in overall longer dwell times for the biased fap compared to the non-biased fbp interpretation of the Necker cube, resulting in different distributions of the respective dwell times (e.g., [14,17]).

### Multistable perception in ASD patients

Only a small number of studies exist about multistable perception in ASD patients. Some studies indicate longer dwell times and thus less reversals in ASD patients (both children and adults) compared to healthy controls during observation of ambiguous figures [22] and of binocular rivalry stimuli [23–25], i.e. when different images are presented to the two eyes and perception alternates spontaneously between the two images (e.g., [26,27]). Other studies found no differences in the reversal dynamics between groups [28,29]. Further, contextual biasing information did not influence adolescents with ASD when they copied ambiguous drawings, whereas a matched control group showed such influence [4]. Finally, there is conflicting evidence whether perceptual dynamics of ambiguous figures and binocular rivalry stimuli correlate with autistic behavioural traits [25,30] or not [28].

The few studies on multistable perception in ASD patients typically used a limited observation time of about one or two minutes [23,24,30] and some of them asked actively for perceptual states during this observation time [22,30]. No study compared distributions of dwell times or variables reflecting the perceptual bias.

In the present study we focused on the perceptual dynamics of patients suffering from Asperger type of autism and a group of matched controls during observation of an ambiguous Necker lattice stimulus [9,10]. The novel approach of the current study is three-fold:

1. Longer presentation durations
  Early studies on the reversal dynamics indicate the existence of a setting phase, which can last up to 3 minutes and during which the reversal rate increases (e.g., [31–33]). We thus presented our ambiguous lattice stimuli for 5 minutes in order to get a more reliable estimate of reversal rates and at the same time more data points to estimate dwell time distributions. Based on results from the earlier studies cited above, we predicted clear evidence for overall less reversals and thus longer dwell times in ASD patients compared to the matched controls.
2. Analysis of dwell time distributions
  One possible explanation for the fap-bias during perception of the Necker stimulus is that people more often adopt from-above-perspectives (fap) than from-below perspectives (fbp) in their every-day lives. This *a priori* perceptual bias can thus be interpreted as strong influence of top-down long-term perceptual memory during the disambiguation of the ambiguous sensory information. It can be quantified by the comparison of the dwell time distributions related to the two perceptual interpretations. Because in Asperger observers the balance between bottom-up sensory information and top-down concepts from memory during perception is described as being shifted towards bottom-up overweighting, we expect a reduced perceptual bias in the Asperger participants compared to healthy controls.
3. Several studies indicate a relation between age and reversal dynamics, which may be due to a change of the weighting between bottom-up and top-down factors over lifetime. Autistic patients show an age-independent preference for small sensory details. Accordingly they should not show such a relation between age and reversal dynamics.

## Methods

### Participants

21 Asperger (AS) participants and 17 healthy control participants were tested in this study. Control participants were selected to match the AS participants in age (± 3 years) and gender. All participants had German school education comparable to junior high school or high school. Due to technical reasons only 19 AS participants (mean age = 41.3, SD = 10.7; 6 females) and 16 controls (mean age = 38.8, SD = 11.5, 6 females) entered the analysis.

All participants completed the autism-spectrum questionnaire “AQ” [34] and the empathy questionnaire “EQ” [35]. In the AQ, AS observers scored above 34 (Mean = 43.1; SD = 5) and the control observers scored below 28 (Mean = 15.1; SD = 5.5). The EQ scores showed the reverse picture—high scores in the control group (Mean = 43.3; SD = 8.2; Min = 29) and low scores in the AS group (Mean = 14.2; SD = 6.3; Max = 28).

All participants had a normal or corrected-to-normal visual acuity. All participants gave their informed written consent. The capacity of all autistic patients to consent had been verified in the context of the psychiatric examination by a consultant psychiatrist. The study was performed in accordance with the ethical standards laid down in the Declaration of Helsinki [36] and was approved by the ethics board of the Albert-Ludwigs-Universität Freiburg, Germany.

### Clinical diagnostics

At the Division of Psychiatry and Psychotherapy, University Medical Center Freiburg, the clinical diagnosis of autism spectrum disorders and AS is established as a consensus diagnosis of a multiprofessional team following the recommendations of the NICE guidelines (National Institute for Health and Clinical Excellence: Autism in Adults: full guideline (https://www.nice.org.uk/guidance/cg142/evidence/full-guideline-pdf-186587677)). “The Guideline development group identified a number of key components that should form the basis of any comprehensive assessment of an adult with possible autism, as follows xx:

- the core symptoms of autism including social-interaction and social-communication difficulties, and stereotypic behaviour
- early developmental history
- behavioural problems
- the impact on current functioning including personal and social functioning, educational attainment and employment
- past and current history of mental and physical disorders
- other neurodevelopmental conditions.

Wherever possible this assessment should be supported by direct observation of the person’s behaviour.” (NICE 2012 page 131).

At the center named above, the diagnostic principles are realized in a structured way. The clinical diagnosis includes a thorough history of the patient following the above principles, a history of carriers (parents, partners, siblings etc.) and behavioral observations in a diagnostic process that usually takes several sessions. Psychometric tools like AQ [34], EQ [35], Australian Scale for Asperger’s Syndrome (ASAS, [37]), SRS [38], BVAQ [39], and BDI [40] are obtained in a routine procedure prior to clinical assessment and are used also for differential diagnostics. Additionally, instruments like ADI-R [41] and ADOS [42] are applied in selected and unclear cases. The same is true for additional neuropsychological tests assessing executive and theory-of-mind capacities. The multiprofessional diagnostic team consists of three experienced senior consultant psychiatrists and two fully qualified senior psychologists. The final consensus diagnosis is made by all persons involved in the diagnostic process, which will invariably include at least two experienced consultant psychiatrists or psychologists.

### Stimuli

We presented Necker lattices (a combination of nine Necker cubes, Fig 1, left) with 5.5° x 6.5° horizontal and vertical visual angle as ambiguous stimuli. The lattice edges were white (173 cd/m^2^) on a dark background (0.3 cd/m^2^). The lattice stimuli were created with a Macintosh G4 computer and displayed on a Philips GD 402 monochrome CRT screen at a refresh rate of 85 Hz.

### Experimental paradigm

An ambiguous Necker lattice (Fig 1, left) was presented continuously for 5 minutes and participants indicated perceived orientation reversals between the from-above-perspective (“fap”, Fig 1 middle) and the from-below-perspective (“fbp”, Fig 1 left) by two different keys. A third key indicated periods with no clear perceptual interpretation.

### Data analysis

**Fig 2:**
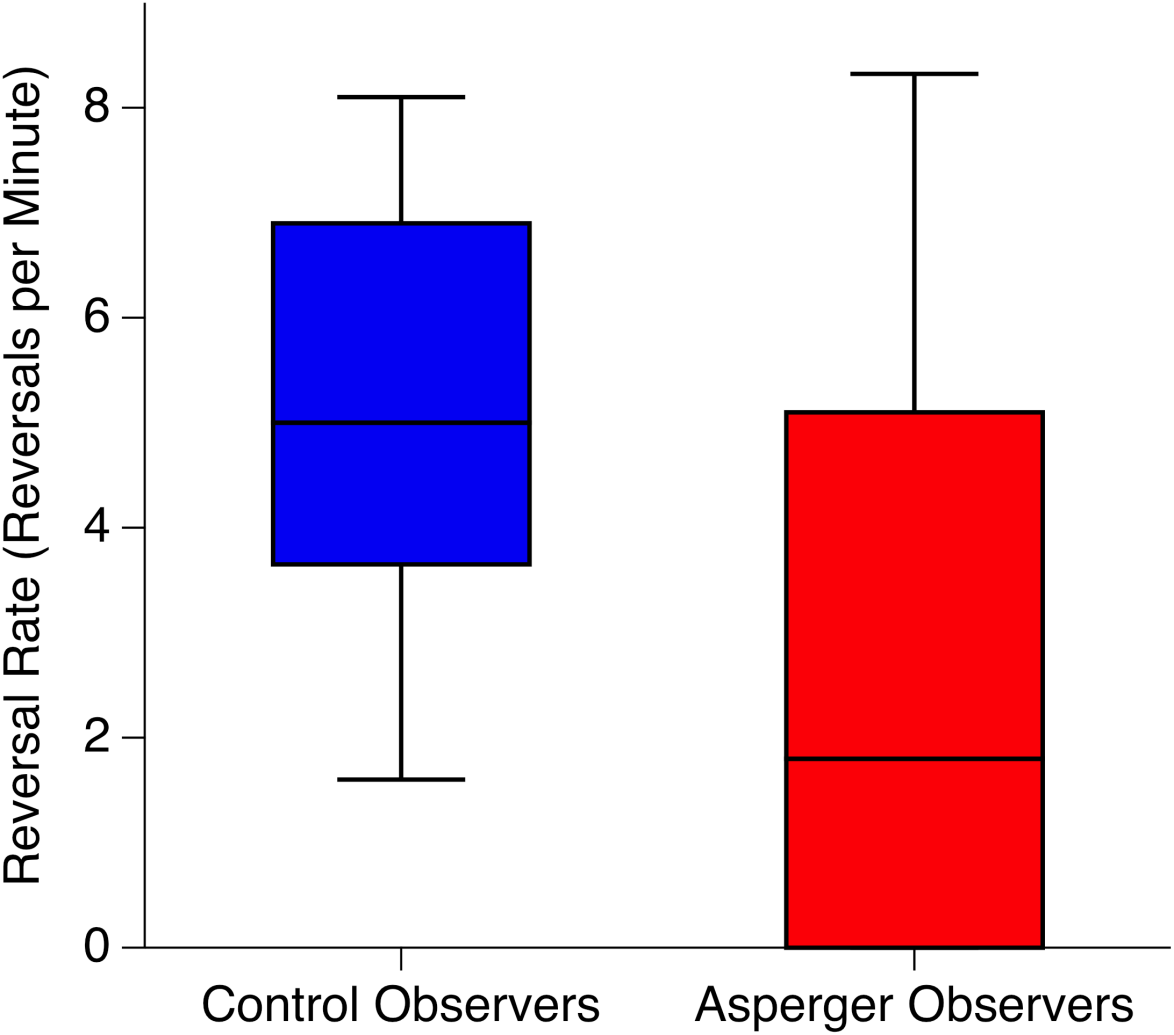
Reversal rates. Median reversal rates (reversals per minute), interquartile range (boxes) and 90%-tile (antennas) for control (blue) and Asperger (red) participants. Notice that the lower quartile and 90-percentile of the Asperger data fall together.

We compared reversal rates (the number of spontaneous perceptual reversals per minute, Fig 2) between observer groups with a Mann-Whitney-U test.

We further calculated separate dwell times (the median durations of temporally stable percepts between reversals) for the two perceptual interpretations (fap and fbp). Reaction times to exogenously induced reversals of disambiguated lattice variants (Fig 1, middle and right figures) are in the range of 500 ms ± 100 ms [10]. We thus took 300 ms as lower bound estimates for dwell times. In rare cases participants pressed two times in succession the same key. This may have happened either by accident or because participants forgot to press a key related to a perceptual reversal. The related data may confound true dwell times and were thus removed before analysis.

**Fig 3:**
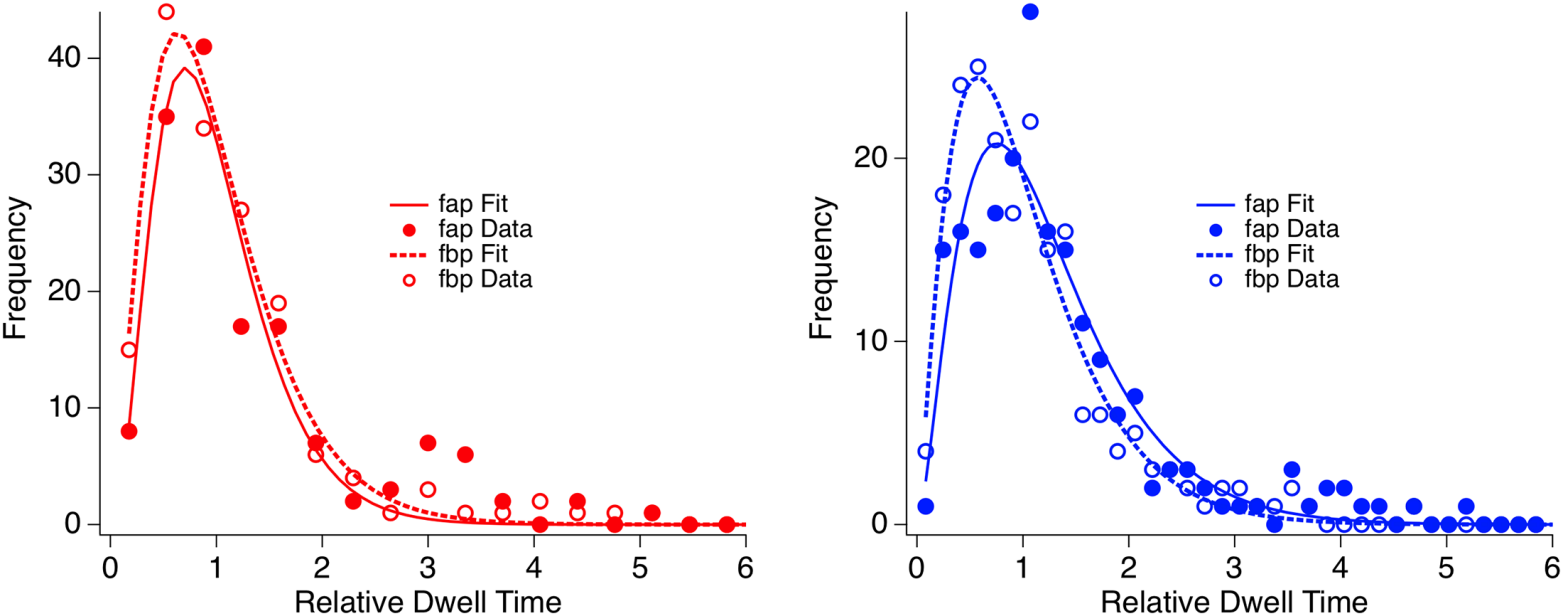
Dwell time distributions. Distributions of the normalized dwell times, separately for the two observer groups (left: ASD patients with red traces and icons; right: control participants with blue traces and icons) and for the two perceptual interpretations. Filled circles correspond to fap (from-above-perspective) data, continuous lines depict the corresponding gamma fits. Open circles correspond to fbp (from-below-perspective), the dotted lines depict the corresponding gamma fits. Differences in ordinate scaling result from the different number of participants and thus data points entering in the two distributions.

It is known that the inter-individual variability in reversal rates (and thus dwell times) is considerably large (e.g., [11]). We removed parts of this variance by dividing the individual dwell times by the individual median dwell time over both perceptual variants. We then compared the normalized median dwell times for the fap interpretation and the fbp interpretation separately for Asperger and control observers by applying Wilcoxon tests. For this analysis participants with less than at least five dwell time values per perceptual interpretation within the 5 min observation period were removed. This resulted in 11 Asperger participants and 14 control participants. In order to visualize differences concerning the perceptual bias, we created distributions and gamma function fits of the cumulated dwell times separately for each perceptual interpretation and group (Fig 3).

Finally, we calculated correlation coefficients between age and reversal rates for the two groups.

## Results

### Reversal rates

We found fewer perceptual reversals for Asperger observers compared to normal controls (p = 0.016, Wilcoxon Test, Fig 2 and Table 1). Particularly, six out of 19 Asperger observers (32%) did not report a single perceptual reversal within the 5 minutes presentation time.

**Table 1:** Perceptual dynamics.

	Control Observers	Asperger Observers
Median Reversal Rates and Interquartile Ranges [reversals per minute]	5 (3.7–6.9)	1.8 (0–5.1)
Median Dwell Times (fap) and Interquartile Ranges [seconds]	3.4 (3.1–4.1)	3.8 (3.3–4.9)
Median Dwell Times (fbp) and Interquartile Ranges [seconds]	3 (2.5–3.4)	3.7 (2.3–5.6)
Number of participants with zero reversals	0	6
Number of participants with undefined percepts	1	5
Total number of periods with undefined percepts	8	20

Five AS-patients and one control person indicated periods of undefined percepts: One Asperger patient indicated one period of an undefined percept, one Asperger observer indicated three, two Asperger observers indicated six and finally one Asperger observer indicated 10 such periods. In contrast only one normal control observer indicated 8 periods of an undefined percept.

### Dwell times

Normal control participants showed significantly longer median dwell times for the fap than for the fbp (p < 0.03, Wilcoxon Test), reflecting the perceptual bias in favour of the fap. No such difference between median dwell times was found for the Asperger patients. This result is supported by Fig 3, which displays the gamma fits of dwell time distributions separately for Asperger (red traces) and control observers (blue traces) and for the two percepts (continuous line: fap; dotted line: fbp). In the case of the normal observers the mode of the gamma fit (peak) from the fap data is at higher dwell times compared to the fbp mode. Further, the fap gamma fit indicates a larger number of longer dwell times than the fbp gamma fit (at higher abscissa values the continuous line is above the dotted line). In the case of Asperger patients, in contrast, the gamma fit traces for the two perceptual interpretations are almost identical.

### Age and reversal rate

**Fig 4:**
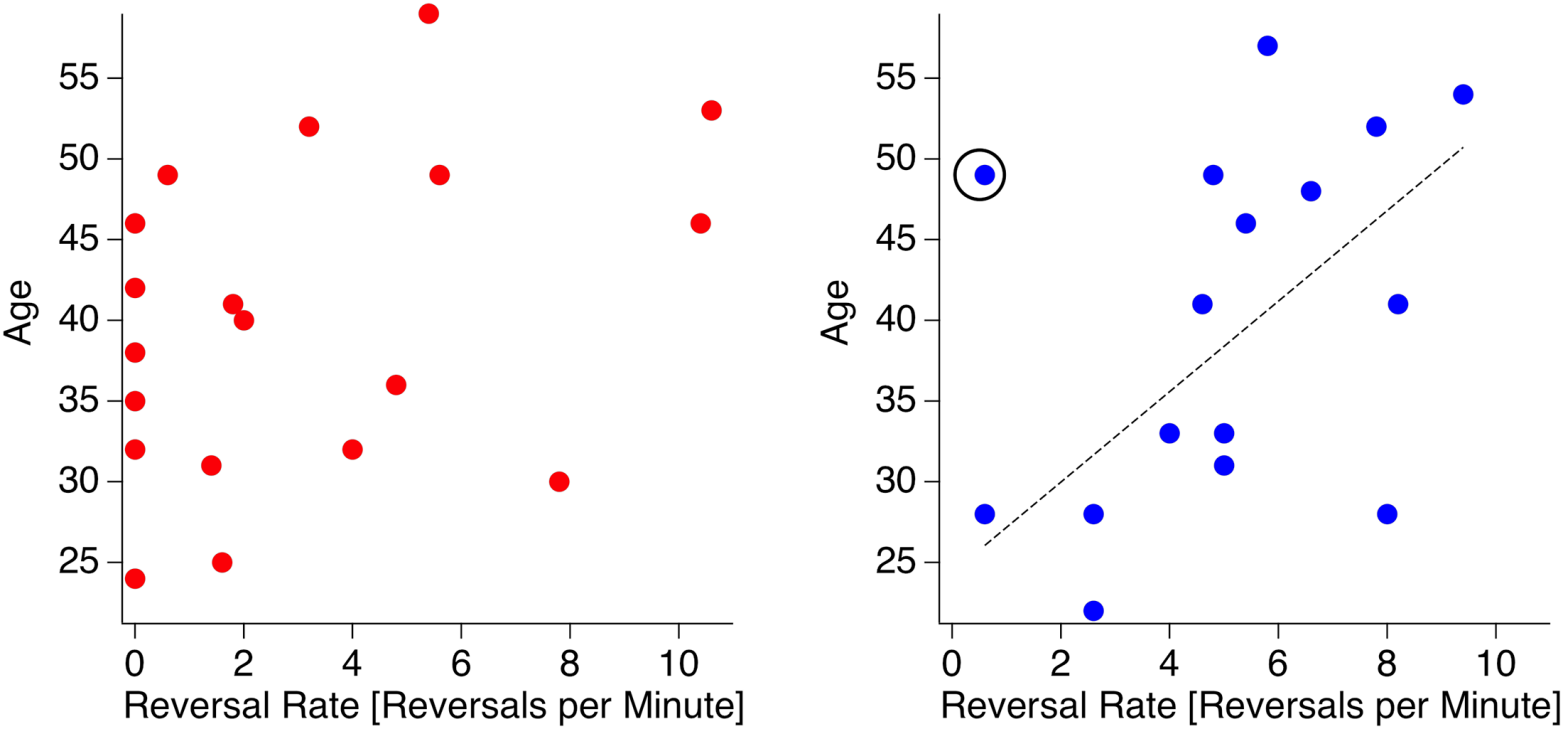
Relation between reversal rate and age. Relation between participants’ age and reversal rates of the Necker lattice. Left: Individual ASD patients in red; right: individual control participants in blue. There is no significant relation between Asperger observers’ age and their reversal rate. Control observers, in contrast, show an increase in reversal rates with age. Dotted lines represent regression lines.

Our control participants show a positive relation between age and reversal rate (Fig 4, right; r_Pearson_ = 0.6 [0.42], p < 0.01 [0.05]; r_Spearman_ = 0.59 [0.44], critical value = 0.52 [0.51]; data in brackets represent the statistics with the outlier, indicated by a black circle). No such relation is visible in the Asperger patients’ data (Fig 4, left).

## Discussion

In the present study Asperger patients and healthy controls observed the ambiguous Necker lattice and indicated perceived orientation reversals by key press. We found fewer reversals in Asperger observers compared to healthy controls. In particular about 32% of the Asperger observers perceived no single reversal within the five minutes observation time, whereas all healthy controls perceived at least three reversals.

In our control participants we found the well-known a priori bias during perception of Necker stimuli with longer dwell times for the from-above-perspective (fap) compared to the from-below-perspective (fbp). No such perceptual bias was observed for the Asperger observers.

Finally, we found more reversals and thus increasing perceptual instability with increasing age of the control participants. No relation between reversal rate and age was observable in the Asperger participants.

### Differences in reversal rates and dwell times between groups

Perceptual interpretations in autistic observers are dominated by small sensory details, whereas they have difficulties to integrate spatial context (e.g., [4]) and prior perceptual experiences (here labelled as perceptual memory, e.g., [5,6]). Evidence for this comes from visual illusion examples, where perceptual memory leads to erroneous interpretations in healthy subjects (e.g., [43]), whereas autistic observers are less susceptible to such illusions [7,8].

We interpret our current results as follows: The a priori incomplete, noisy and to varying degrees ambiguous sensory information is weighted with memorized concepts in order to construct stable and reliable percepts. During observation of an ambiguous stimulus, like the Necker lattice in our experiment, the exogenous physical information stays constant during observation. Several theoretical approaches thus assume endogenous neural fluctuations (“endogenous noise”) and a crossing of a certain threshold every now and then may cause perceptual alternations between the different interpretations [44–46].

Perception in Asperger observers is characterized by an underweight of endogenous perceptual memory factors, and an overweight of exogenous sensory input. They thus seem to see the world more “objectively”, which may be disadvantageous in many situations but it may sometimes also be advantageous [47,48].

During observation of the ambiguous Necker lattice, this weight imbalance may result in a reduction or even elimination of the long-term memory bias in our Asperger observers. Further, endogenous noise may also be less influential during perception of the Necker lattice in Asperger compared to control observers, which may result in the observed difference in reversal rates.

### Age-related change of the reversal dynamics in the literature

Several studies on age-related differences in the perceptual dynamics of ambiguous figures report either a negative correlation between reversal rates and age [49,50] or an inverted-U pattern, i.e. positive correlation for early ages and negative correlation for older ages [51,52]. The data in the current study, in contrast, point to more reversals with increasing age. How can this discrepancy be explained?

Of course, one important factor is the different age ranges of the participants between studies. Another important factor may be the observation time of the ambiguous figures, which was below two minutes in all the above-cited studies: Early work on the reversal dynamics during perception of ambiguous figures indicate the existence of a setting phase, which can last up to 3 minutes and during which the reversal rate increases (e.g., [31–33]). It may well be possible that the duration of such an initial setting phase may increase with age. The influence of the setting phase, and particularly its duration, on reversal rates is larger, the shorter the observation sequence is. We thus presented our ambiguous lattice stimuli for 5 minutes in order to get a more reliable estimate of reversal rates and at the same time more data points to estimate dwell time distributions.

### Age-related change of the reversal dynamics in the present study

At the time point of birth, we have little perceptual memory. Perception is thus almost entirely based on what enters through our senses. During our lives there is a continuous accumulation of perceptual experiences, many of them stored in our memories. At the same time the efficacy of our sensory organs decreases with age (e.g. cataract, senile miosis, [53]). Based on these considerations we can assume that the weighting between sensory evidence and memorized perceptual concepts changes over lifetime from an initial overweight of exogenous factors to an overweight of endogenous factors with increasing age. Concurrently the impact of endogenous fluctuations during Necker lattice perception may be amplified with increasing age, resulting in less stable percepts and thus an increasing reversal rate.

We currently postulate that autistic persons do not (or to a lesser degree) underlie the age-related change of weighting between bottom-up and top-down factors during perception, as observed in healthy participants. As a consequence they don’t show the correlation between reversal rate and age. Of course, this hypothesis has to be further tested in subsequent experiments.

## Summary and outlook

Our findings of lower reversal rates and an absence of the bias during perception of the Necker lattice are in agreement with the observed dominance of bottom-up over top-down influence during the perceptual process in patients with autism spectrum disorder. A newly developed experimental paradigm that uses ambiguous figures and disambiguated versions in healthy observers, allowed us recently to quantify top-down contributions of long-term and shorter-term memory to the perceptual disambiguation [19,54]. As a next step we will apply this paradigm to our Asperger observers in order to look for quantitative estimates to the qualitative findings from the present study.

Our findings concerning the correlation between observers’ age and their reversal dynamics are of particular interest for the following reasons. Asperger patients seem to be less affected by this kind of age related modification of perception. Aging of our sensory organs is the most often discussed explanation for age-related changes in perception, however, this can be ruled out for the present effect, because Asperger patients’ senses underlie the same aging processes as healthy controls. This is another interesting topic for further research.

In the current study we used the geometric Necker lattice as an ambiguous figure, where ambiguity concerns the interpretation of figure depth. We expect the same results with the simpler Necker cube, but this has to be shown in a separate study. Further, there are other well-known ambiguous figures, where ambiguity concerns different visual categories, like motion (e.g., von Schiller’s Stroboscopic Alternative Motion display, [55]) or more semantic aspects, like Boring’s Old/Young Woman stimulus [56]. One of our next steps is thus to study the multistable perception dynamics of patients with different types and complexity levels of stimulus ambiguity, e.g. concerning the emotional expression of a face [57].

Overall, our results indicate that the topic of multistable perception provides new insights into patterns of altered perception in autistic patients. At the same time such patient studies provide new insights concerning basic mechanisms of visual perception, like cognitive aspects of perceptual aging.

## Supporting information

S1 File Reversal rate and stability duratin raw data. “FB” = lattice front side bottom right;
“FT” = lattice front side top left;
line 4: Header labeling the individual participants with "GX_RR" or "FX_RR": "G" = control participants; "F" = Asperger patients; "X" substitute for letters from "A" to "V", coding individual participants in the two groups;
“RR”: shortcut for “reversal rates”;
line 5: individual reversal rate values from 5 minutes Necker lattice observation;
line 8: "GX_FB" or "GX_FT" or "FX_FB" or "FX_FT": "GX" and "FX" coding of the individual participants from the control and Asperger groups, as explained above; "FT": header for stability duration of the front-side top left lattice percept; "FB": header for stability duration of the front-side bottom right lattice percept; subsequent rows (9ff): stability duration data in milliseconds.
(XLSX)

## References

[1] von Helmholtz H. Handbuch der physiologischen Optik. Hamburg/Leipzig: Leopold Voss; 1911.

[2] Kersten D, Mamassian P, Yuille A. Object perception as Bayesian inference. Annu Rev Psychol. 2004;55: 271–304. 10.1146/annurev.psych.55.090902.142005 14744217

[3] Kornmeier J, Wörner R, Riedel A, Bach M, Tebartz van Elst L. A Different View on the Checkerboard? Alterations in Early and Late Visually Evoked EEG Potentials in Asperger Observers. Gilbert S, editor. PLoS ONE. 2014;9: e90993 10.1371/journal.pone.0090993 24632708 PMC3954585

[4] Allen ML, Chambers A. Implicit and explicit understanding of ambiguous figures by adolescents with autism spectrum disorder. Autism. 2011;15: 457–72. 10.1177/1362361310393364 21486897

[5] Frith U. Autism: Explaining the Enigma. Oxford, United Kingdom: Blackwell Publishing; 2003.

[6] Mitchell P, Ropar D. Visuo-spatial Abilities in Autism: A Review. Inf Child Dev. 2004;13: 185–198.

[7] Happe FG. Studying weak central coherence at low levels: children with autism do not succumb to visual illusions. A research note. J Child Psychol Psychiatry. 1996;37: 873–7. 8923230 10.1111/j.1469-7610.1996.tb01483.x

[8] Simmons DR, Robertson AE, McKay LS, Toal E, McAleer P, Pollick FE. Vision in autism spectrum disorders. Vision Res. 2009;49: 2705–39. 10.1016/j.visres.2009.08.005 19682485

[9] Kornmeier J, Heinrich SP, Atmanspacher H, Bach M. The reversing “Necker Wall”–a new paradigm with reversal entrainment reveals an early EEG correlate. ARVO 2001 Annual Meeting. 2001. p. 409.

[10] Kornmeier J, Bach M. Early neural activity in Necker-cube reversal: Evidence for low-level processing of a gestalt phenomenon. Psychophysiology. 2004;41: 1–8. 10.1016/j.visres.2004.10.006 10.1016/j.visres.2004.10.006 14692995

[11] Borsellino A, De Marco A, Allazetta A, Rinesi S, Bartolini B. Reversal time distribution in the perception of visual ambiguous stimuli. Kybernetik. 1972;10: 139–144. 5021011 10.1007/BF00290512

[12] Long GM, Toppino TC. Enduring interest in perceptual ambiguity: alternating views of reversible figures. Psychol Bull. 2004;130: 748–68. 10.1037/0033-2909.130.5.748 15367079

[13] Brascamp JW, van Ee R, Pestman WR, van den Berg AV. Distributions of alternation rates in various forms of bistable perception. J Vis. 2005;5: 287–298. 10.1167/5.4.1 15929652

[14] Wernery J, Atmanspacher H, Kornmeier J, Candia V, Folkers G, Wittmann M. Temporal processing in bistable perception of the Necker cube. Perception. 2015;44: 157–168. 10.1068/p7780 26561969

[15] Kornmeier J, Bach M. Ambiguous figures—what happens in the brain when perception changes but not the stimulus. Front Hum Neurosci. 2012;6: 1–23. 22461773 10.3389/fnhum.2012.00051 PMC3309967

[16] Kersten D, Yuille A. Bayesian models of object perception. Curr Opin Neurobiol. 2003;13: 150–8. 12744967 10.1016/s0959-4388(03)00042-4

[17] Murata T, Matsui N, Miyauchi S, Kakita Y, Yanagida T. Discrete stochastic process underlying perceptual rivalry. Neuroreport. 2003;14: 1347–52. 10.1097/01.wnr.0000077553.91466.41 12876471

[18] Dobbins AC, Grossmann JK. Asymmetries in Perception of 3D Orientation. He S, editor. PLoS ONE. 2010;5: e9553 10.1371/journal.pone.0009553 20209050 PMC2832009

[19] van Rooij M, Atmanspacher H, Kornmeier J. Hysteresis in Processing of Perceptual Ambiguity on Three Different Time Scales. In: Papafragou A, Grodner D, Mirman D, Trueswell J, editors. Proceedings of the 38th Annual Conference of the Cognitive Science Society. Boston, USA; 2016. pp. 568–573.

[20] Wexler M, Duyck M, Mamassian P. Persistent states in vision break universality and time invariance. Proc Natl Acad Sci. 2015;112: 14990–14995. 10.1073/pnas.1508847112 26627250 PMC4672830

[21] Mamassian P, Landy MS. Observer biases in the 3D interpretation of line drawings. Vision Res. 1998;38: 2817–2832. 9775328 10.1016/s0042-6989(97)00438-0

[22] Sobel DM, Capps LM, Gopnik A. Ambiguous figure perception and theory of mind understanding in children with autistic spectrum disorders. Brit J Dev Psychol. 2005;23: 159–174.

[23] Robertson CE, Kravitz DJ, Freyberg J, Baron-Cohen S, Baker CI. Slower Rate of Binocular Rivalry in Autism. J Neurosci. 2013;33: 16983–16991. 10.1523/JNEUROSCI.0448-13.2013 24155303 PMC3807027

[24] Freyberg J, Robertson CE, Baron-Cohen S. Reduced perceptual exclusivity during object and grating rivalry in autism. J Vis. 2015;15: 11 10.1167/15.13.11 26382002 PMC4594764

[25] Karaminis T, Lunghi C, Neil L, Burr D, Pellicano E. Binocular rivalry in children on the autism spectrum: Binocular rivalry in autism. Autism Res. 2017;10: 1096–1106. 10.1002/aur.1749 28301094 PMC5485021

[26] Blake R. A Primer on Binocular Rivalry, Including Current Controversies. Brain Mind. 2001;2: 5–38.

[27] O’Shea RP, Kornmeier J, Roeber U. Predicting visual consciousness electrophysiologically from intermittent binocular rivalry. Ward LM, editor. PLoS ONE. 2013;8: e76134 10.1371/journal.pone.0076134 24124536 PMC3790688

[28] Said CP, Egan RD, Minshew NJ, Behrmann M, Heeger DJ. Normal binocular rivalry in autism: Implications for the excitation/inhibition imbalance hypothesis. Vision Res. 2013;77: 59–66. 10.1016/j.visres.2012.11.002 23200868 PMC3538943

[29] Intaitė M, Georgescu A, Noreika V, von Saldern MAO, Vogeley K, Falter CM. Adults with autism spectrum disorders (ASD) have atypical perception of ambiguous figures when bottom-up and top-down interactions are incongruous. Revis. 10.1177/1362361318782221 30288989

[30] Best CS, Moffat VJ, Power MJ, Owens DG, Johnstone EC. The boundaries of the cognitive phenotype of autism: theory of mind, central coherence and ambiguous figure perception in young people with autistic traits. J Autism Dev Disord. 2008;38: 840–7. 10.1007/s10803-007-0451-8 18004653

[31] Brown KT. Rate of apparent change in a dynamic ambiguous figure as a function of observation time. Am J Psychol. 1955;68: 358–371. 13248970

[32] Price JR. Studies of reversible perspective: A methodological review. Behav Res Methods Instrum. 1968;1: 102–106. 10.3758/BF03206959

[33] Price JR. Effect of extended observation on reversible perspective duration. Psychon Sci. 1969;16: 75–76.

[34] Baron-Cohen S, Wheelwright S, Skinner R, Martin J, Clubley E. The autism-spectrum quotient (AQ): evidence from Asperger syndrome/high-functioning autism, males and females, scientists and mathematicians. J Autism Dev Disord. 2001;31: 5–17. 11439754 10.1023/a:1005653411471

[35] Baron-Cohen S, Wheelwright S. The empathy quotient: an investigation of adults with Asperger syndrome or high functioning autism, and normal sex differences. J Autism Dev Disord. 2004;34: 163–75. 15162935 10.1023/b:jadd.0000022607.19833.00

[36] World Medical Association. Declaration of Helsinki: ethical principles for medical research involving human subjects. JAMA. 2000;284: 3043–3045. 10.1001/jama.284.23.3043 11122593

[37] Melfsen S, Walitza S, Attwood A, Warnke A. Validierung der deutschen Version der Australian Scale of Asperger’s Syndrome (ASAS). Z Für Kinder- Jugendpsychiatrie Psychother. 2005;33: 27–34. 10.1024/1422-4917.33.1.27 15714838

[38] Constantino JN, Davis SA, Todd RD, Schindler MK, Gross MM, Brophy SL, et al Validation of a brief quantitative measure of autistic traits: comparison of the social responsiveness scale with the autism diagnostic interview-revised. J Autism Dev Disord. 2003;33: 427–433. 12959421 10.1023/a:1025014929212

[39] Vorst HC., Bermond B. Validity and reliability of the Bermond?Vorst Alexithymia Questionnaire. Personal Individ Differ. 2001;30: 413–434. 10.1016/S0191-8869(00)00033-7

[40] Beck AT, Ward CH, Mendelson M, Mock J, Erbaugh J. An inventory for measuring depression. Arch Gen Psychiatry. 1961;4: 561–571. 13688369 10.1001/archpsyc.1961.01710120031004

[41] Lord C, Rutter M, Le Couteur A. Autism Diagnostic Interview-Revised: a revised version of a diagnostic interview for caregivers of individuals with possible pervasive developmental disorders. J Autism Dev Disord. 1994;24: 659–685. 7814313 10.1007/BF02172145

[42] Lord C, Risi S, Lambrecht L, Cook EH, Leventhal BL, DiLavore PC, et al The autism diagnostic observation schedule-generic: a standard measure of social and communication deficits associated with the spectrum of autism. J Autism Dev Disord. 2000;30: 205–223. 11055457

[43] Gregory R. What are illusions? Perception. 1996;25: 503–4. 10.1068/p250503 8865293

[44] Moreno-Bote R, Rinzel J, Rubin N. Noise-induced alternations in an attractor network model of perceptual bistability. J Neurophysiol. 2007;98: 1125–39. 10.1152/jn.00116.2007 17615138 PMC2702529

[45] Gigante G, Mattia M, Braun J, Del Giudice P. Bistable Perception Modeled as Competing Stochastic Integrations at Two Levels. Friston KJ, editor. PLoS Comput Biol. 2009;5: e1000430 10.1371/journal.pcbi.1000430 19593372 PMC2700962

[46] Braun J, Mattia M. Attractors and noise: twin drivers of decisions and multistability. Neuroimage. 2010;52: 740–51. 10.1016/j.neuroimage.2009.12.126 20083212

[47] Pellicano E, Burr D. When the world becomes “too real”: a Bayesian explanation of autistic perception. Trends Cogn Sci. 2012;16: 504–10. 10.1016/j.tics.2012.08.009 22959875

[48] Koldewyn K, Jiang YV, Weigelt S, Kanwisher N. Global/Local Processing in Autism: Not a Disability, but a Disinclination. J Autism Dev Disord. 2013;43: 2329–2340. 10.1007/s10803-013-1777-z 23378063 PMC3679259

[49] Heath HA, Orbach J. Reversibility of the Necker cube: IV. Responses of the elderly people. Percept Mot Skills. 1963;17: 625–626. 10.2466/pms.1963.17.2.625 14057288

[50] Aydin S, Strang NC, Manahilov V. Age-related deficits in attentional control of perceptual rivalry. Vision Res. 2013;77: 32–40. 10.1016/j.visres.2012.11.010 23206550

[51] Holt GL, Matson JL. The effects of age on perceptual changes using two new perspectives of the Necker cube. Bull Psychon Soc. 1976;8: 4–6. 10.3758/BF03337055

[52] Patel K, Reed M. Multistable Perception in Older Adults: Constructing a Whole from Fragments. Brain Sci. 2016;6: 10 10.3390/brainsci6010010 27011204 PMC4810180

[53] Greenlee MW, Sekuler AB, editors. Visual perception and visual cognition in healthy and pathological ageing [Internet]. Frontiers Media SA; 2014 10.3389/978-2-88919-253-3 PMC4018523 24834059

[54] Liaci E, Fischer A, Heinrichs M, Tebartz van Elst L, Kornmeier J. Mona Lisa’s happiness is up to 34% in the beholder’s eye –relations between emotional and geometric ambiguity. submitted;

[55] Schiller PV. Stroboskopische Alternativversuche. Psychol Forsch. 1933;17: 179–214. 10.1007/BF02411959

[56] Boring EG. A new ambiguous figure. Am J Psychol. 1930;42: 444–445. 10.2307/1415447

[57] Liaci E, Fischer A, Heinrichs M, van Elst LT, Kornmeier J. Mona Lisa is always happy–and only sometimes sad. Sci Rep. 2017;7: 43511 10.1038/srep43511 28281547 PMC5345090

